# Using High-Dimensional Approaches to Probe Monocytes and Macrophages in Cardiovascular Disease

**DOI:** 10.3389/fimmu.2019.02146

**Published:** 2019-09-12

**Authors:** Sarah A. Dick, Rysa Zaman, Slava Epelman

**Affiliations:** ^1^University Health Network, Toronto General Research Institute, Toronto, ON, Canada; ^2^Ted Rogers Centre for Heart Research, Toronto, ON, Canada; ^3^Peter Munk Cardiac Center, Toronto, ON, Canada

**Keywords:** macrophages, monocytes, cardiovascular, scRNA-seq, myocardial infarction, atherosclerosis

## Abstract

High dimensional approaches that characterize single cells at unprecedented depth have helped uncover unappreciated heterogeneity, a better understanding of myeloid cell origins, developmental relationships and functions. These advancements are particularly important in cardiovascular disease, which remains the leading cause of death worldwide. Gradual, monocyte-dependent inflammatory processes, such as the development of atherosclerotic plaque within arterial vessels, contrasts with the robust acute response within the myocardium that occurs when a vessel is occluded. Monocytes and macrophages differentially contribute to tissue injury, repair and regeneration in these contexts, yet many questions remain about which myeloid cell types are involved in a coordinated, organ-level sterile inflammatory response. Single cell RNA sequencing, combined with functional analyses have demonstrated that at least three populations of resident cardiac macrophages exist, and after tissue injury, there is significant diversification of the tissue macrophage pool driven by recruited monocytes. While these studies have provided important insights, they raise many new questions and avenues for future exploration. For example, how do transcriptionally defined sub-populations of cardiac macrophages relate to each other? Are they different activation states along a pre-defined trajectory of macrophage differentiation or do local microenvironments drive newly recruited monocytes into distinct functions? The answers to these questions will require integration of high-dimensional approaches into biologically relevant *in vivo* experimental systems to ensure the predicted heterogeneity possess a functional outcome.

## Background

Mononuclear phagocytes are central mediators of cardiovascular (CV) disease, the leading cause of death worldwide (1). In broad terms, CV disease can be classified into two forms; ischemic and non-ischemic. Ischemic injury initiates within coronary arteries, with gradual accumulation of LDL cholesterol in the artery wall over decades, leading to a smoldering, monocyte-dependent chronic inflammatory response that drives atherosclerotic plaque expansion. Acute plaque rupture leads to diminished blood flow to a segment of the myocardium (myocardial infarction), resulting in cell death with or without reperfusion injury—both processes that also trigger substantial monocyte recruitment. Non-ischemic cardiovascular injury represents a heterogeneous group of etiologies that include hemodynamic strain (hypertension), inflammatory myocarditis (infectious or autoimmune), cardiotoxicity (such as from chemotherapy), as well as a variety of other factors, all of which also trigger monocyte recruitment (2). After injury, cardiac contractile function can be impaired, promoting the development of heart failure. Importantly, ischemic and non-ischemic etiologies both trigger recruitment of monocytes from circulation and activate resident macrophages that live within the tissue—which together, coordinate the inflammatory and reparative response to injury.

The traditional view for decades has been that a monocyte produced in the bone marrow enters tissue and becomes a tissue macrophage in health and disease (3). This concept, while initially important—overlooked substantial heterogeneity within both monocyte production, monocyte fate after entry into tissue—and separately, the heterogeneity within resident tissue macrophages. Recent technical advancement in genetic fate mapping, multi-dimensional (single-cell mass cytometry [CyTOF], and novel flow cytometric markers; ~40 markers) and high-dimensional approaches (i.e., single cell RNA sequencing [scRNA-seq]; 1,000–5,000 transcripts), represents a key inflection point in our ability to probe the mononuclear phagocyte system. Subsequent computational analyses can not only help functionally separate closely related cell types in an unbiased fashion but can infer developmental relationships between cells. In this review, we will define our current understanding of monocyte and macrophage heterogeneity in CV disease (heart and vasculature), where limitations exist, and possible opportunities for future investigation in the context of using high-dimensional approaches.

## Circulating Monocyte Heterogeneity

During development monocytes are produced in the fetal liver (through erythroid myeloid progenitors that migrate from the yolk sac) and subsequently from definitive hematopoietic stem cells (HSCs) (4). After birth, definitive HSCs in the bone marrow become the major source of monopoiesis. Blood monocytes, derived from common myeloid progenitor cells, are first produced as Ly6C^hi^ monocytes (CD14^+^CD16^−^ in humans), which are referred to as classical/inflammatory monocytes due to their ability to extravasate into tissues, where they execute a variety of effector functions following injury. In addition, Ly6C^hi^ monocytes may differentiate into macrophages or dendritic cells depending on the local tissue environment, or they persist as a monocyte subset and exit tissue, as demonstrated in the lung (5). In patients, increased numbers of intermediate CD14^+^CD16^+^ monocytes have been correlated to increased risk of CV disease, impaired recovery after myocardial infarction, microvascular dysfunction and worse clinical outcomes (6–9).

**Figure 1:**
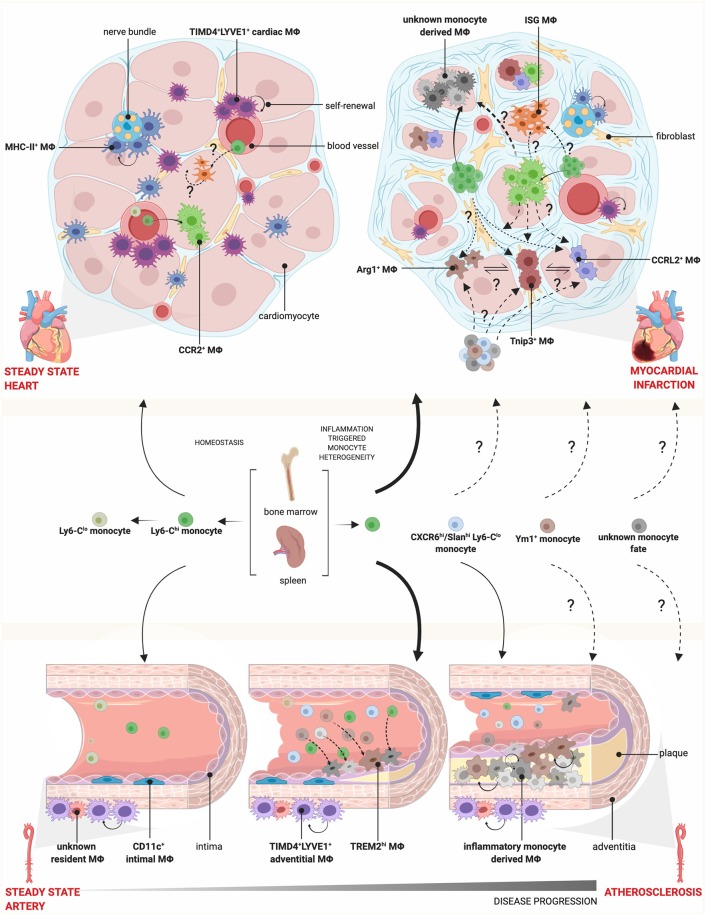
Monocyte and macrophage heterogeneity in steady state and cardiovascular disease. During homeostasis, Ly6C^hi^ monocytes circulate through blood vessels and infiltrate tissue, where they give rise to CCR2^+^ MΦs, while Ly6C^lo^ monocytes patrol the vasculature. Cardiac MΦs are further composed of monocyte-independent self-renewing TIMD4^+^LYVE1^+^ and MHC-II^+^ resident MΦs, which localize preferentially near blood vessels and nerve bundles, respectively. During myocardial infarction, there is increased monopoiesis and release of Ly6C^hi^ monocytes from the spleen and bone marrow, which are recruited to the injured heart and give rise to diverse MΦ subsets. Whether these MΦ subsets are a spectrum of activation states or arise via pre-defined monocyte fates, such as Ym1^+^ or CXCR6^hi^/Slan^hi^ Ly6C^lo^ monocytes as identified in other disease models, is not known. Conversely, there is a loss of TIMD4^+^LYVE1^+^ and MHC-II^+^ resident MΦs. In the vessels, the intima is lined with CD11c^+^ MΦs and the adventitia contain TIMD4^+^LYVE1^+^ MΦs and other undefined resident MΦ populations. In atherosclerosis, TREM2^hi^ MΦs and inflammatory monocyte-derived MΦs accumulate in the intima, expand via self-renewal and participate in plaque growth. How this fate is defined and the contribution of CXCR6^hi^/Slan^hi^ Ly6C^lo^ monocytes, found in the circulation of patients correlating with disease severity, is unknown. Mϕ, macrophage.

Examination of chromatin accessibility within the genome of Ly6C^hi^ monocytes has led to the prediction that differentiation from classical to Ly6C^lo^ non-classical monocytes (through an intermediate stage) is the default pathway (10). Non-classical Ly6C^lo^ monocytes (CD14^lo^CD16^+^ in humans) play an important role in patrolling the vasculature and maintaining vessel wall integrity (11). With the use of scRNA-seq, several groups have attempted to uncover further heterogeneity that exists within the blood monocyte pool at steady-state. These studies suggest a heterogeneous population of intermediate monocytes (murine Ly6C^int^ monocytes and human CD14^+^CD16^+^ monocytes) (10, 12). Given the heterogeneous nature of intermediate monocytes, variation between individual human donors and different single cell technologies, it is not surprising that some studies failed to demonstrate a defined intermediate population (13). Whether increased intermediate monocytes are a marker of systemic processes driving increased cardiac pathology, or whether CD14^+^CD16^+^ intermediate monocytes are themselves infiltrating the myocardium and promoting pathology, has yet to be determined. Advances in profiling circulating monocytes using CyTOF have yielded enticing clues about potential novel monocyte subsets that may arise in patients with coronary artery disease, including increased CXCR6 and Slan (6-sulfo-LacNac) expression on non-classical monocytes correlating with increasing severity of atherosclerosis (14) (Figure 1). While a detailed and unbiased single cell approach focused on peripheral monocytes (and other circulating cells) has yet to be undertaken in CV disease, we have compelling evidence in animal studies that in the setting of inflammation, novel monocyte subsets are liberated from the bone marrow, which may have important functional implications.

## Monocyte Diversity Triggered During Inflammation, Aging, and Implications for CV Disease

During cardiac injury, the monocytic demand is beyond that available in circulation and in bone marrow or splenic reservoirs. This HSC drive leads to the increased production and mobilization of myeloid cells, a process termed “emergency hematopoiesis” (15). The bone marrow senses increased stress at distant sites through soluble factors, such as GM-CSF and IL-1β (16, 17). In the setting of myocardial infarction, a subset of CCR2^+^CD150^+^CD48^−^ hematopoietic progenitors with enhanced proliferative capacity are mobilized in the bone marrow through an *Mtg16*-dependent process (18). Deletion of *Mtg16* decreased monocyte production and led to impaired infarct healing. Additionally, myocardial infarction and risk factors for myocardial infarction (sleep deprivation) induces a state of stress—which itself can trigger increased bone marrow HSC activity and promote development of atherosclerotic lesions (19). The chronic months-long process of atherosclerosis progression in mice (decades in humans) vs. the very acute inflammatory and hemodynamic fluctuations of a myocardial infarct trigger very different hematopoietic responses—and while little is known about the types of monocytes produced in both settings, it is tempting to speculate.

For example, scRNA-seq revealed a “neutrophil-like” Ly6C^hi^ monocyte subset that was mobilized in response to LPS injection and contained increased expression of granule enzyme myeloperoxidase protein indicating an enhanced direct pathogen killing function (20). Additionally, SatM monocytes (segregated-nucleus-containing atypical monocytes) were found to be responsible for fibrosis, but not inflammation, in the setting of pulmonary fibrosis (21). A Ym1^+^Ly6C^hi^ monocyte population has been recently shown to be liberated from the bone marrow to the colon during the resolution phase of colitis (22). Thus, early production of monocytes skewed toward inflammation or fibrosis may be balanced by later production of monocytes that promote tissue repair.

The accumulation of somatic mutations in hematopoietic stem and progenitor cells as we age can lead to the clonal expansion of a particular hematopoietic founder cell, termed clonal hematopoiesis, due to its competitive advantage over others. The role of clonal hematopoiesis in cardiovascular disease has recently emerged, contributing to the aberrant accumulation of inflammatory monocyte-derived macrophages in atherosclerosis, hypertension, and ischemic injury (23, 24). Similarly, increased proliferation and expansion of hematopoietic stem and progenitor cells in the *Apoe*^−/−^ mouse fed a high fat diet led to the development of atherosclerotic lesions (25). This suggests that excessive myelopoiesis is not only a consequence of the inflammatory injury response but when not properly regulated, can promote/exacerbate disease progression. Although conflicting results have been gleaned from clinical trials using anti-inflammatory drugs in ischemic and non-ischemic heart disease (26), a recent clinical trial demonstrated a beneficial role for the IL-1β inhibitor Canakinumab, resulting in decreased cardiovascular events in patients with atherosclerosis (27). This effect has been attributed to its potential ability to blunt excessive hematopoiesis and monocyte production; however, macrophages within advanced atherosclerotic plaque expand numerically through local proliferation rather than continual monocyte recruitment (28), thereby suggesting alternative mechanisms may also be involved.

## Macrophage Heterogeneity in Steady State

Several groups, using a combination of genetic fate mapping and single cell transcriptomics have defined three populations of tissue macrophages within the myocardium that are distinct in origin, monocyte-dependence, and function (29–36) (Figure 1). TIMD4^+^LYVE1^+^MHC-II^lo^CX3CR1^lo^ macrophages (termed TIMD4^+^LYVE1^+^ macrophages) represent an embryonically-derived subset that renews almost entirely through *in situ* proliferation without significant blood monocyte input in adult animals, downregulating CX3CR1 as animals age (29, 32). A portion of TIMD4^+^LYVE1^+^ macrophages upregulate MHC-II and lose expression of TIMD4 and LYVE1 (termed MHC-II^+^ macrophages); which renew *in situ*, but also receive measurable, albeit minimal monocytes in adult animals. Lastly, a numerically smaller population of CCR2^+^MHC-II^hi^ macrophages exists, which is continuously replaced by monocyte-derived cells. Sex-mismatched heart transplant recipients confirm the peripheral blood origin of CCR2^+^ cardiac macrophages in humans (33).

Both CCR2^+^ and MHC-II^+^ macrophages can process and present antigen to T-cells, however their definitive role during homeostasis is unclear. Analogous populations have been reported to be associated with nerve bundles (34) and it is possible that they suppress inflammation at these sites. Resident macrophages are found in the atrioventricular node of the myocardium, and when depleted, conduction abnormalities can be detected–which suggests they may also reinforce efficient electrical conduction (37). LYVE1^+^ macrophages are found closely associated with the vasculature, promote endothelial cell activation, patterning of coronary vasculature and are efficient in the uptake of apoptotic cell material (29–31, 34). Depletion of resident macrophages in steady state induced development of cardiac fibrosis (34), which together suggest multiple important homeostatic functions.

A fourth population of tissue cardiac macrophages has now been identified through scRNA-seq in the uninjured myocardium, increasing in number after injury (32, 38, 39). This population is characterized by a strong interferon stimulated gene signature (termed ISG MFs). Whether ISG macrophages represent a unique tissue macrophage subset or are part of a spectrum of activation states is unclear. Moreover, their role in homeostasis is also unknown, which highlights the need to develop tools to isolate and study this novel population. Importantly, in the setting of myocardial infarction, blockade of the type I interferon response enhanced infarct recovery suggesting a critical role for this pathway (and possibly this subset) in adverse LV remodeling (38).

A variety of approaches have been used to study monocytes and macrophages within blood vessels, with a focus on the aorta as a surrogate of coronary vasculature. Both embryonic-derived macrophages, and neonatal macrophages, contribute to aortic macrophage composition. ScRNA-seq has been used to examine macrophage heterogeneity within the aorta, with a focus on the atherosclerotic environment (see below). A resident macrophage signature was seen within a single cluster in naïve mice that expressed *Lyve1*, with gene expression similarities to *Lyve1*/*Timd4* expressing cardiac macrophages, however additional heterogeneity within the total macrophage population was not assessed (40, 41). This population of arterial LYVE1^+^ macrophages resides in the arterial adventitia and is maintained locally via self-renewal though interaction with the vasculature smooth muscle cells (42).

## Diversification of Cardiac Macrophage Populations in Ischemic Injury

In the setting of ischemic injury, the injured myocardium recruits Ly6C^hi^ monocytes in large numbers—an observation established by numerous groups. The parallel fates of resident macrophages and recruited monocytes at single cell resolution is less clear. Genetic fate mapping and scRNA-seq reveal that resident TIMD4^+^LYVE1^+^ and MHC-II^hi^ macrophages are lost within the ischemic zone, presumably due to cell death. Recruited monocytes appear to have two principle paths after tissue entry. The first, observed both in acute, and sub-acutely after infarct involves a unique trajectory relative to the resident macrophage population, characterized by multiple unique transcriptional states (32, 39). The transition from Ly6C^hi^ monocytes to early macrophages tightly correlates with upregulation of hypoxia-inducible genes (*Hif1a, Vegfa*, etc), upregulation of mature macrophage genes (*Mertk*) and downregulation of monocyte genes (*Ly6c2*). ScRNA-seq has been performed at day 3, 4, 7, and 11 post-infarct by different groups, demonstrating unique transcriptional identities that become more clear over time (32, 36, 39). Importantly, key findings from different studies support the loss of the resident cardiac macrophage subpopulations, and in parallel–recruitment of monocytes that begin to specify as early as day 3-4, and subsequently their differentiation into a variety of transcriptionally unique populations, including those with more reparative properties.

Secondly, a subset of recruited monocyte-derived macrophages developed overlapping transcriptional identities that were nearly identical to resident macrophages (32). Interestingly, these recruited macrophages did not upregulate a handful of lineage-specifying genes such as *Lyve1* and *Timd4*, which proved useful as cell surface markers to reliably track the original resident macrophage populations without the need for genetic fate mapping. Despite the near transcriptional identity between these recruited macrophage populations, depletion of only resident macrophages resulted in decreased cardiac function and adverse remodeling, suggesting either a functionally (or temporally) non-redundant role. While precise functioning of resident macrophages in this context is unclear, it may be due to their ability to modulate the fate of recruited monocytes. Depletion of tissue resident macrophages increased the number of two recruited monocyte-derived macrophage fates (termed ARG1^+^ and CCRL2^+^ macrophages) (33). This sheds new light on the diversity of monocyte-derived macrophages within the injured heart and highlights tissue resident macrophages as important orchestrators of monocyte fate specification.

Not all resident macrophage populations behave similarly post-ischemic injury. For example, the depletion of CCR2^+^ cardiac macrophages prior to ischemic injury (43) reduced the number of pathologic ISG macrophages (IFIT3^+^) and increased the number of ITGB7^+^ macrophages. We do not yet know what the function is of ITGB7^+^ cardiac macrophages, however their gene expression profile suggests they could be reparative. It is also unclear whether individual resident tissue macrophage subpopulations (TIMD4^+^ vs. CCR2^+^) directly influence recruited monocytes, or have the capacity to recruit monocytes that have been shown to have a direct reparative role in other models [Ym1^+^Ly6C^hi^ monocytes (22)]. It is equally likely that the depletion of individual resident macrophage populations changes the microenvironment rather than acting directly on recruited monocytes, and thus the altered microenvironment directs monocyte fate decisions after monocytes enter tissue.

## Diversification of Aortic Macrophage Populations in Atherosclerosis

Although much is known about the role of monocytes in the formation of atherosclerosis [as reviewed in (44)], two recent reports have now shown via scRNA-seq the heterogeneity that exists within the immune cell compartment of atherosclerotic lesions in two independent mouse models fed a high fat diet (*Apoe*^−/−^ and *Ldlr*^−/−^) (40, 41). Consistent with both studies, is the identification of monocytes within atherosclerotic aortas (*Ly6c2, Ccr2*). Beyond the resident macrophage population seen in control and atherosclerotic mice (*Lyve1, Pf4*), inflammatory macrophages and TREM2^hi^ macrophages were also demonstrated, the latter being enriched in pathways linked to lipid metabolism and calcification (41) (Figure 1). Although macrophage populations were consistent between healthy and diseased aortas, the number of macrophages were increased, as well as the expression of a number of genes implicated in lipid and cholesterol metabolism and oxidative stress. In one study, the authors evaluated earlier (11 weeks) vs. more advanced atherosclerosis (20 weeks) with little difference in macrophage heterogeneity. This is consistent with the observation that lesional macrophages accumulate through local proliferation (28). These initial experiments proved to be informative, however it was not possible to differentiate macrophages isolated from within the artery wall itself (intimal) vs. those that accumulate outside the wall in the surrounding adventitia. Given the very different microenvironments in these two regions, it will be important for future studies to separately investigate each region. Moreover, atherosclerosis tends to develop regionally in the aorta (near the aortic root, lesser curvature of the aortic arch), thus understanding the differences between regions at the single cell level could provide clues to the regional nature of atherosclerosis initiation and progression.

## Outstanding Questions and Conclusions

One key outstanding question which remains is how to interpret heterogeneity revealed by single cell data and move forward with functional studies. Retrospectively identifying population clusters bioinformatically is only the first step. Building a differentiation map of infiltrating monocytes and prospectively sorting populations based on robust combinations of surface markers will be an important approach to characterize individual populations. As technology and computational approaches improve, it will be important to integrate single cell mapping with tissue localization. For example, it is possible to perform single cell transcriptomics using methods that preserve tissue localization [MERFSIH, SlideSeq (45, 46)], whereby monocyte fate can be mapped from the blood vessel lumen to varied anatomical niches found within ischemic or atherosclerotic tissue by tracking individual or groups of RNAs. Linking single cell transcriptomic advancements to single cell epigenetics and proteomics will further enhance resolution (47, 48). The analysis of the comprehensive landscape of cells within tissues has led to the generation of whole mouse and human cell atlas projects which take a relatively broad approach to cell characterization, but highlights the strength of this technology to be able to compare cells across tissues and species (49, 50). These multi-disciplinary approaches require collaboration, given the wide breadth of skills required to integrate different technologies. The use of single cell technologies to assess immune cells come with limitations, such as differential extraction of individual subsets and reduced read depth compared to bulk techniques, which are caveats that must be acknowledged. In addition, most of the initial studies utilized single replicates, thus the reproducibility of individual data sets is still a major question in the field. The identification of new subsets of monocytes and monocyte-derived cells within tissues at steady state and inflammation already highlights the profound role single cell technologies have had revealing previously unknown heterogeneity. Future insights into their function could allow for better therapeutic targets that aim to hinder chronic inflammation while promoting tissue repair and regeneration.

## Author Contributions

SD and SE wrote the manuscript. RZ created the figure with BioRender.com.

### Conflict of Interest Statement

The authors declare that the research was conducted in the absence of any commercial or financial relationships that could be construed as a potential conflict of interest.
